# Micro-RNA content of circulating extracellular vesicles in early rheumatoid arthritis as biomarkers and mediators of methotrexate efficacy

**DOI:** 10.1093/rheumatology/kead569

**Published:** 2023-11-01

**Authors:** Daniel Maunder, Philip M Brown, Ben Barron-Millar, Dennis W Lendrem, Najib Naamane, Jamie Macdonald, Xiao N Wang, John D Isaacs, Amy E Anderson, Ann W Morgan, Rachel E Crossland, Sarah L Mackie, Arthur G Pratt

**Affiliations:** Translational and Clinical Research Institute, Newcastle University, Newcastle Upon Tyne, UK; Translational and Clinical Research Institute, Newcastle University, Newcastle Upon Tyne, UK; Musculoskeletal Unit, Newcastle upon Tyne Hospitals NHS Foundation Trust, Newcastle Upon Tyne, UK; Translational and Clinical Research Institute, Newcastle University, Newcastle Upon Tyne, UK; Translational and Clinical Research Institute, Newcastle University, Newcastle Upon Tyne, UK; Translational and Clinical Research Institute, Newcastle University, Newcastle Upon Tyne, UK; Translational and Clinical Research Institute, Newcastle University, Newcastle Upon Tyne, UK; Translational and Clinical Research Institute, Newcastle University, Newcastle Upon Tyne, UK; Translational and Clinical Research Institute, Newcastle University, Newcastle Upon Tyne, UK; Musculoskeletal Unit, Newcastle upon Tyne Hospitals NHS Foundation Trust, Newcastle Upon Tyne, UK; Translational and Clinical Research Institute, Newcastle University, Newcastle Upon Tyne, UK; School of Medicine, University of Leeds, Leeds, UK; Leeds Biomedical Research Centre, Leeds Teaching Hospitals NHS Trust, Leeds, UK; Translational and Clinical Research Institute, Newcastle University, Newcastle Upon Tyne, UK; School of Medicine, University of Leeds, Leeds, UK; Leeds Biomedical Research Centre, Leeds Teaching Hospitals NHS Trust, Leeds, UK; Translational and Clinical Research Institute, Newcastle University, Newcastle Upon Tyne, UK; Musculoskeletal Unit, Newcastle upon Tyne Hospitals NHS Foundation Trust, Newcastle Upon Tyne, UK

**Keywords:** rheumatoid arthritis, microRNA, DMARDs, biomarkers

## Abstract

**Objectives:**

Extracellular vesicles (EVs) are abundant in body fluids, contributing to intercellular signalling by transferring cargo that includes microRNAs (miRs)—themselves implicated in pathobiology. For the first time we evaluated the potential of EV miRs to contribute diagnostic information in early RA, predict methotrexate (MTX) efficacy or shed light on the drug’s mechanism of action.

**Methods:**

Seven hundred and ninety-eight miRs isolated from serum-derived EVs of 46 patients with untreated RA, 23 with untreated polymyalgia rheumatica (PMR; inflammatory disease control group) and 12 in whom significant inflammatory disease had been excluded (non-inflammatory controls; NICs) were profiled (NanoString); the same measurements were made for RA patients after 6 months’ MTX treatment. Analyses took multiple testing into account.

**Results:**

Twenty-eight EV miRs were robustly differentially expressed between early RA (but not PMR) patients and NICs after correction for age and sex, suggesting discriminatory value. Cross-validated partial least squares-discriminant analysis also indicated the predictive potential of a distinct baseline EV miR signature with respect to MTX-induced remission at 6 months. The change in expression of 13 miRs over the course of MTX treatment differed significantly between responders and non-responders, and four of those exhibiting increased relative abundance amongst responders have known roles in regulating the pathogenic potential of synovial fibroblasts, namely miR-212-3p, miR-338-5p, miR-410-3p and miR-537.

**Conclusion:**

Our data highlight the potential of serum EV miRs as diagnostic and therapeutic biomarkers, highlighting a novel potential mechanism by which MTX may exert its therapeutic effect in early RA that warrants further investigation.

Rheumatology key messages

- Circulating extracellular vesicle microRNA profiles may help diagnose or predict therapeutic response in early RA.
- EV microRNAs that restrain disease-promoting properties of synovial fibroblasts increase with methotrexate-induced remission in RA.

## Introduction

RA is a common and sometimes disabling inflammatory rheumatic disease resulting from immune-mediated destruction of synovial joints. Outcomes are improved by early and effective suppression of inflammation [1], but diagnostic uncertainty continues to hamper prompt initiation of DMARDs for some patients. Methotrexate (MTX) remains the most widely administered first-line DMARD for new-onset RA, and pooled trial data indicate 40% of patients experience at least a 50% improvement in disease activity on this drug, with some achieving remission [2]. However, determination of its efficacy—and hence the need for alternative therapy in non-responders—is possible only months after initiation, contributing to sustained joint inflammation for many. The availability of non-invasive biomarkers predictive of efficacy would enable rational personalization of treatment decisions at the point of diagnosis, at the same time informing mechanisms by which MTX exerts its therapeutic effect in this heterogeneous disease. One such mechanism, upon whose importance several lines of evidence now converge, is the interruption of purine processing to potentiate cellular accumulation and release of adenosine [3]. Beyond direct paracrine immunomodulatory effects that follow, MTX-induced enhancement of adenosine signalling may have indirect benefits in RA by driving release of extracellular vesicles (EVs) [4]. EVs are a class of lipid bilayer particles that are secreted into the extracellular space by nearly all cells; they are a rich source of signalling molecules, whose function as vehicles for transferring their cargo between cells and tissues is increasingly appreciated [5].

MicroRNAs (miRs) are small non-coding RNAs that regulate gene translation through 3′UTR binding [6]. There is an increasing body of evidence suggesting they too play a role in the pathogenesis of immune-mediated inflammatory diseases (IMIDs) including early RA [7], but, due in part to the challenges in harmonizing assays for readouts subject to considerable temporal fluctuation, their attractiveness as a tractable biomarker source has been questioned. Indeed, it has been suggested that the miR content of circulating EVs may offer a more stable resource [8]. Whilst they have been shown to modulate immune responses in RA through IL-1 and TNF signalling, relatively little is known about EV miR makeup [9]. Indeed, EVs represent a rich source of potential biomarkers, and their prevalence in blood, serum and urine make them easy to access in a routine clinical setting [10–12]. To address the need for optimizing reproducible methodology for the development of EV miR measurement platforms, we recently developed a simple but robust protocol for profiling of low-concentration EV RNA using the NanoString Technologies nCounter^®^ platform [13]. Careful characterization of miRs in circulating serum EVs during the earliest phase in the natural history of RA and its treatment therefore provides a timely means to explore whether serum EV miR expression discriminates patients with newly presenting, untreated RA, predicts their therapeutic response to MTX, or is differentially regulated according to therapeutic response. This backdrop provides the motivation for our study.

## Methods

### Patient volunteers

Consecutive patients ≥16 years of age were enrolled from two clinics. RA patients were from the Northeast Early Arthritis Cohort (NEAC) where they (i) were DMARD and corticosteroid naïve at the time of enrolment (topical/inhaled corticosteroids permitted), (ii) fulfilled 2010 ACR/EULAR diagnostic criteria for RA, also being assigned a clinical diagnosis of the condition by their consulting rheumatologist, and (iii) were commenced on oral MTX as a first-line DMARD [14, 15]. Detailed clinical evaluations and blood draws for research were undertaken at baseline and an intended follow-up time point of 6 months, at which point individuals who had achieved clinical remission (defined as a disease activity score based on assessment of 28 peripheral joints and CRP; DAS28-CRP), without the need for systemic steroid therapy in the preceding 2 months, were classified as having achieved ‘MTX-induced remission’ (henceforth ‘responders’, as opposed to ‘non-responders’). Concomitant initiation of hydroxychloroquine and/or an intramuscular glucocorticoid bolus were permitted at the time of enrolment providing this followed baseline blood draw, but individuals prescribed oral corticosteroids and/or alternative DMARDs at baseline were excluded. Second, patients referred from primary care with suspected polymyalgia rheumatica (PMR) were enrolled from Leeds Teaching Hospitals NHS Trust (LTHT); these individuals were also DMARD and corticosteroid naïve at enrolment. Detailed clinical evaluation accompanied research blood draw at inception. Thereafter, suspected PMR patients were classified according to whether or not they fulfilled criteria for PMR; amongst those that did not, individuals with alternative immune-mediated rheumatological diagnoses were excluded, leaving a comparator group of ‘non-inflammatory controls’ (NICs). All patients provided written, informed consent to participate in the study, which was approved by the Newcastle and North Tyneside or Camberwell St Giles Research Ethics Committees, UK (REC references 12/NE/0251 and 13/LO/1094, respectively).

### Serum EV isolation, miR extraction and quality control

Blood drawn into serum separator tubes (SST^TM^, BD Biosciences, San Jose, CA, USA) at either site underwent centrifugation (1200 *g*, 7 min), serum supernatant being aliquoted and frozen at −80°C within 4 h of blood draw. Upon defrosting, a protocol previously established and validated in our laboratory for high quality EV miR isolation from biological fluid was deployed as described [13], with morphology and size distribution of EVs characterized using validated methods [16] (additional detail provided in Supplementary Data S1, available at *Rheumatology* online).

### MicroRNA quantification using NanoString nCounter technology

Serum EV miR expression profiling was performed using the nCounter Human v3 miRNA Expression Assay kit (NanoString Technologies, Seattle, WA, USA) and NanoString nCounter FLEX platform, as previously described [17], incorporating 5 µl of RNA samples and a 24 h hybridization time. The code set comprises 98% of miR sequences found in miRbase v22 and includes 798 mature miRs, six positive and eight negative controls, six ligation controls and five reference controls.

### Data processing and analysis

Raw NanoString output (RCC) files were first subject to standard quality control (QC) procedures including imaging, binding density and positive control linearity QC using nSolver software version 4.0 (NanoString Technologies). One RA follow-up sample was thereby excluded for purposes of downstream analysis due to a ‘ligation’ QC flag. For those samples that passed nSolver QC, raw counts were then exported for downstream processing and analysis using the DESeq2 package [18], implemented in the R statistical environment (R Foundation for Statistical Computing, Vienna, Austria) [19]. Here, for each contrast, geometric mean normalization was first performed using the ‘DESeq’ function, with exclusion of low-count miRs by independent filtering as part of the ‘results’ function; probes included/excluded in analyses using this approach are listed in Supplementary Table S1, available at *Rheumatology* online. Differential expression testing was then applied to filtered probes.

Descriptive statistics were used to determine differences between clinical characteristics of comparator groups. For cross-sectional analyses contrasting miR expression between independent samples, potential confounding clinical variables were accounted for by incorporating them as co-variates in DESeq formulae; differentially expressed miRs were then prioritized using the Wald test (α = 5%) corrected for false discovery using the Benjamini–Hochberg method [20]. Partial least squares discriminatory analysis (PLS-DA) with internal model validation (5-fold cross-validation method [21]) was used to discriminate responders and non-responders by baseline miR expression. Changes (delta) in EV miR expression between baseline and follow-up visits in responders and non-responders were determined by linear modelling and adjustments made for age, sex and baseline expression, using the lm() function in R. Others have reported both age and sex differences in response to treatment: men are more likely to respond to treatment, and older patients less likely to respond [22–27]. Accordingly, potential miRs were prioritized by identifying those with clinically plausible, statistically significant interactions with age, sex, or age by sex effects. Functional analysis of differentially expressed or regulated miR lists generated during aforementioned analyses was undertaken with reference to publicly available resources, including the online miR target-prediction tools ‘miRDB’ [28] and ‘mirPath’ with the ‘microT-CDS’ algorithm. [29, 30].

## Results

### Patients and sample quality control

Peripheral blood samples were processed from 46 patients with newly presenting RA, recruited from NEAC, and 35 patients referred to the ADDRESS-PMR Study at LTHT, of whom 23 acquired a confirmed PMR diagnosis during specialist assessment and 12 were determined ‘non-inflammatory’ controls (NIC) for the purpose of our study. A representative electron micrograph of visualized vesicles isolated from a single serum sample is depicted in Supplementary Fig. S1A (available at *Rheumatology* online); nanoparticle tracking analysis with a NanoSight LM10 microscope (five captures) indicated a mode EV size of 175 nm, corresponding to the expected range for EVs (Supplementary Fig. S1B, available at *Rheumatology* online). Baseline clinical characteristics of participants are summarized in Table 1, from which it may be observed that early RA patients were significantly younger in age than PMR patients and NICs, as expected; in addition, a higher percentage of NIC patients were female compared with RA patients (83% *vs* 57%), although this difference was not significant; these findings informed the selection of potential confounders to include as co-variates in downstream cross-sectional analyses. Finally, measured acute phase markers were significantly higher amongst patients in RA and PMR ‘inflammatory control’ groups than in the NIC group, as predicted.

**Table 1.** General characteristics of all patients in study

	Diagnosis			*P*-value
	RA (*n* = 46)	PMR (*n* = 23)	NIC (*n* = 12)	
Age, median (IQR), years	58 (49.3–68.8)	76 (70.5–79.5)	66 (59–72.5)	<0.05
Female, *n* (%)	27 (57.5)	16 (69.6)	10 (83.3)	ns
CRP, median (IQR), mg/l	10.5 (5–20.5)	28.0 (12.0–55.5)	3.0 (3–8.0)	<0.05
ESR, median (IQR), mm/h	22.0 (8.3–32.8)	28.5 (21.5–45.8)	16.0 (6.5–24)	<0.05

*P*-values calculated using Kruskal–Wallis test for continuous variables and chi-square test for categorical variables. IQR: interquartile range; NIC: non-inflammatory control; ns: not significant; PMR: polymyalgia rheumatica.

Amongst the 46 RA patients whose samples are described in Table 1 and who were commenced on MTX at baseline, robust follow-up data and paired serum for EV miR extraction was available for a total of 41 individuals, the median time between baseline and follow-up sampling/clinical assessment being 18.6 weeks (range: 13–31 weeks). Of these, 14 were classified as responders and the remaining 27 as non-responders based on pre-determined criteria (see Methods); their baseline characteristics are summarized in Table 2, from which it may be observed that comparator groups were matched except in terms of baseline disease activity; here, in keeping with typical reports in the literature, the DAS28 of individuals who subsequently entered disease remission was significantly lower than that of those who did not—again informing co-variates selection for downstream analyses.

**Table 2.** Demographic and clinical data for early RA patients according to MTX response (after median 18.6 weeks’ treatment; see text)

	Methotrexate outcome		*P*-value
	Responders (*n* = 14)	Non-responders (*n* = 27)	
Age, median (IQR), years	55 (50.3–67.5)	58 (50–70)	ns
Female, *n* (%)	10 (71.4)	13 (48.1)	ns
CRP, median (IQR), mg/l	6 (4–14.5)	14 (7–39)	ns
ESR, median (IQR), mm/h	13.5 (5–22.5)	28 (9.5–37.5)	ns
Baseline DAS28-CRP, median (IQR)	3.37 (3.13–3.87)	4.92 (3.98–5.64)	<0.05
RF-positive, number (%)	10 (71.4)	15 (55.6)	ns
CCP-positive, number (%)	10 (71.4)	15 (55.6)	ns
Time interval, median (IQR), months	4.4 (3.8–4.9)	4.4 (3.6–4.6)	ns

Time interval calculated by time between baseline and follow-up sample donation. *P*-values calculated using Kruskal–Wallis test for continuous variables and chi-square test for categorical variables. DAS28-CRP: disease activity score based on assessment of 28 peripheral joints and CRP; IQR: interquartile range; MTX, methotrexate; ns: not significant.

### A serum EV miR ‘signature’ discriminates early RA patients

**Figure 1.**
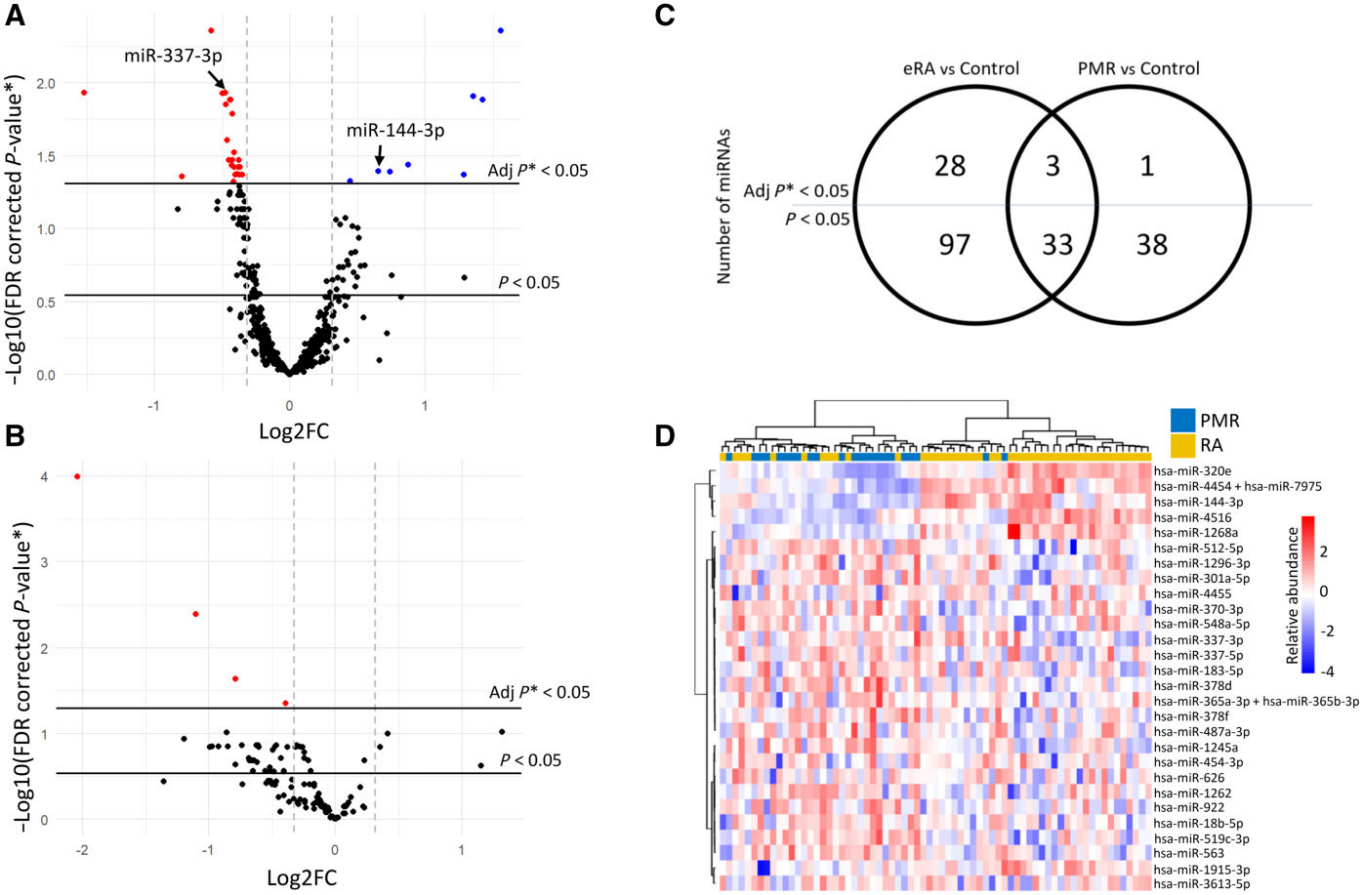
An EV miR signature for discriminating untreated early RA from control groups. (**A** and **B**) Volcano plots displaying miRs significantly downregulated (red; upper left) and up-regulated (blue; uppper right) in RA (**A**) or PMR (**B**) compared to NIC control group. Each data point is a single miR and horizontal lines represent significance thresholds for α = 5% (Wald test) with and without multiple test correction. Vertical lines represent a fold change of ±1.25. (**C**) Venn diagram summarizing the number of significantly differentially expressed miRs between both disease states and disease controls according to whether the findings are or are not robust to multiple test correction (above and below horizontal line, respectively; see Supplementary Table S2 and S3, available at *Rheumatology* online for complete listings). (**D**) Heatmap displaying *Z*-scores for 28 uniquely RA-associated miRs. Direct comparison between RA and PMR groups. Clustering by Euclidian distance. *Adj *P*: adjusted *P*-value employing false discovery rate (FDR); EV: extracellular vesicle; FC: fold change; miR: microRNA; PMR: polymyalgia rheumatica

The DESeq2 package (Wald test) was deployed in R to identify miRs differentially expressed between members of the three diagnostic categories summarized in Table 1, incorporating potential confounding clinical variables (baseline age and sex) as co-variates. First, we established that 130 out of 798 miRs, measured using the nCounter Human v3 miRNA Expression Assay kit, were differentially expressed in treatment-naïve RA patients compared with NIC individuals, of which 31 were robust to multiple test correction (Fig. 1A; the 1.25-fold differential expression cut-off reflected data dimensionality in this exploratory analysis). A full list of differentially expressed EV miRs is presented in Supplementary Table S2, available at *Rheumatology* online. We next conducted a similar comparison of treatment-naïve patients diagnosed with an *alternative* IMID typically associated with a high acute phase response, PMR, with the same NIC group—reasoning that any miRs found to be differentially expressed in *both* contrasts were likely a consequence of systemic inflammation, rather than disease-specific phenomena. Seventy-one miRs were found to be differentially expressed in this comparison; just four were robust to multiple test correction (Fig. 1B and Supplementary Table S3, available at *Rheumatology* online) of which three overlapped between comparisons, namely miR-150-5p, miR-342-3p and miR-3641-5p. Fig. 1C depicts a Venn diagram summarizing these contrasts and highlighting 28 miRs uniquely deregulated in early, untreated RA. The dendrogram depicted in Fig. 1D shows the results of unsupervised clustering of RA and PMR patients according to EV miR expression, confirming the potential discriminatory value of such a ‘signature’ for RA in this context. Intriguingly, amongst the r28 miRs signature, miR-337-3p, which was downregulated in RA compared with controls, has several predicted target genes that are implicated in RA pathogenesis, namely *JAK2* and *STAT3* [31] and *IL13RA1* [32]. Amongst miRs significantly upregulated in RA was miR-144-3p, which has previously been shown to be associated with pathological inflammation [33]. Kyoto Encyclopedia of Genes and Genomes (KEGG) pathway analysis using the online target prediction tool ‘mirPath’ with the ‘microT-CDS’ algorithm showed that the 28 uniquely deregulated miRs in RA were associated with TGF-β signalling, which has previously been implicated in the activation of synovial fibroblasts in RA [34, 35], more significantly than any other pathway (Supplementary Table S4, available at *Rheumatology* online).

### Potential of serum EV miR content for predicting methotrexate response in early RA

**Figure 2.**
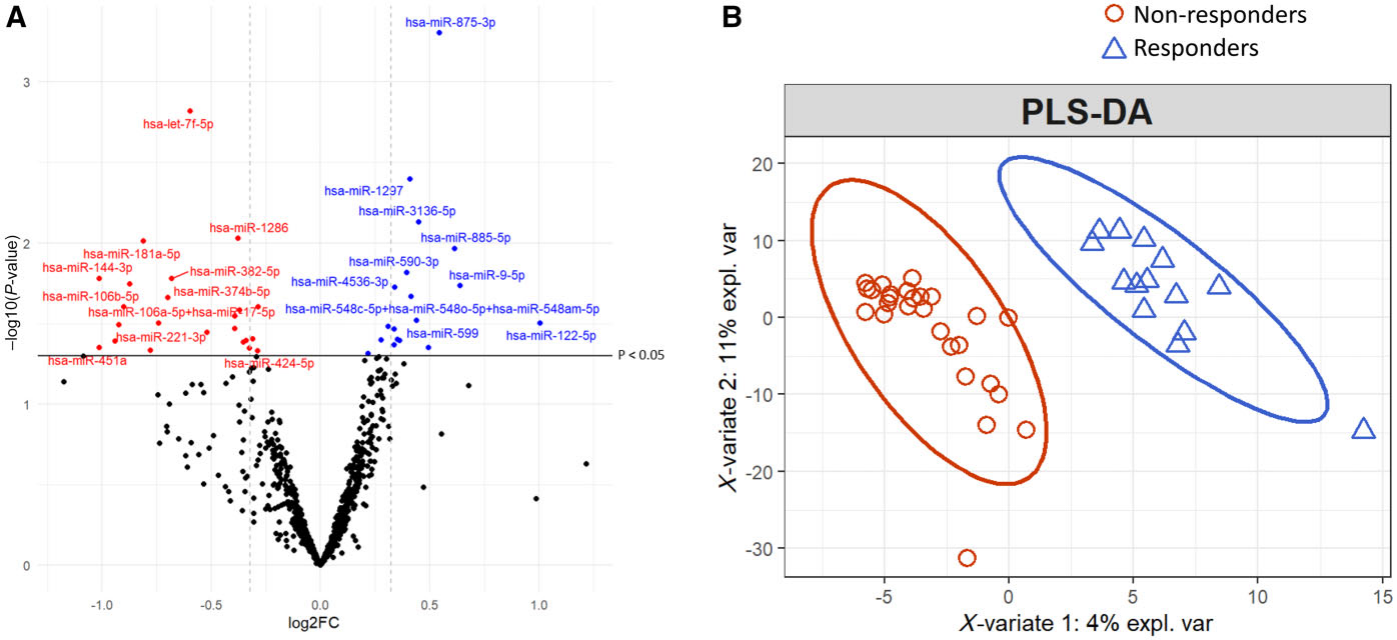
A pre-treatment EV miR signature for predicting early methotrexate response in newly diagnosed RA. (**A**) Volcano plots displaying miRs significantly downregulated (red) and up-regulated (blue) at baseline in RA patients who responded to MTX compared with those who did not. Each data point is a single miR and horizontal lines represent nominal significance threshold for α = 5% (Wald test; no multiple test correction). Vertical lines represent a fold change of ±1.25. (**B**) PLS-DA plot demonstrating statistical difference between MTX responders (orange) and non-responders (blue) at baseline. EV: extracellular vesicle; FC: fold change; miR: microRNA; MTX: methotrexate; PLS-DA: partial least squares discriminatory analysis

Focusing only on the 41 RA patients in our study for whom robust follow-up clinical information was available at a median of 18 weeks following MTX initiation (Table 2), we next used the same methodology to test for differential expression between baseline miR counts for early responders *vs* non-responders to this intervention according to a clinically relevant definition of remission without the need for systemic steroids in the preceding 2 months (see Methods). As a potentially confounding factor, baseline DAS28 was incorporated into the analysis as a co-variate. Although 41 miRs were shown to be differentially expressed at baseline between subsequent responders *vs* non-responders, none of these were robust to multiple test correction (Fig. 2A;  Supplementary Table S5, available at *Rheumatology* online). KEGG pathway analysis again showed a strong association between these 41 differentially expressed miRs and TGF-β signalling (full list of pathways shown in Supplementary Table S6, available at *Rheumatology* online). Reasoning that this was likely attributable to a limited sample size, a PLS-DA approach with 5-fold cross-validation was deployed to identify any overall difference in baseline serum EV miR expression between responders and non-responders that might motivate downstream validation work. Whilst often used as a predictive tool, PLS-DA can also be used for descriptive modelling and, as indicated in Fig. 2B, this analysis highlighted a striking difference between comparator groups.

### Dynamic changes in serum EV miR abundance following methotrexate commencement are consistent with a synovial fibroblast-mediated mechanism of clinical response

**Figure 3.**
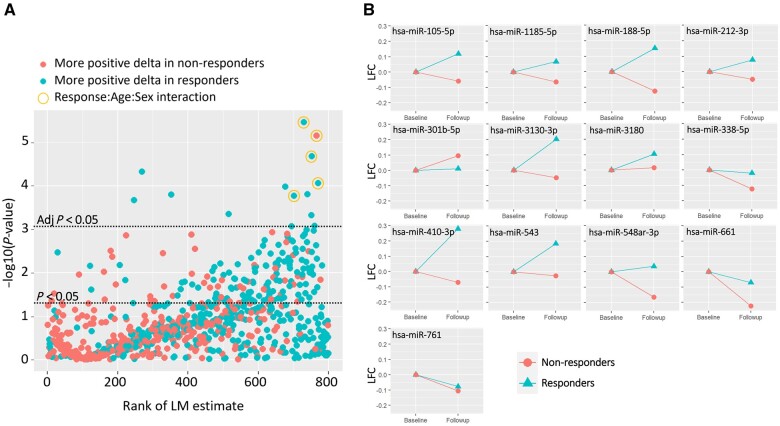
Differential dynamic changes in EV miR expression according to methotrexate responsiveness. (**A**) Scatter plot displaying linear model output testing responder effect for all 798 miRs included in the analysis. Negative log-transformed *P*-values are plotted against the rank of the size-of-effect estimate for each miR. Each data point is a single miR and horizontal lines represent significance thresholds for α = 5% with and without multiple test correction for effect difference between responders and non-responders, with the direction of difference represented by colour as indicated in the key. Response-regulated miRs subject to age: sex interactions (see text) are circled. (**B**) Plots displaying log fold-change pre- and post-treatment for the 13 miRs for which there was a significantly different change in abundance over time between responders and non-responders. EV: extracellular vesicle; LFC: log fold change; LM: linear model; miR: microRNA

We next evaluated whether the dynamics of EV miR expression over time differed significantly between early MTX responders and non-responders. Linear models were generated for each of the 798 miRs to interrogate the relationship between response to MTX and the change in miR expression from baseline to follow-up, including age, sex, miR expression at baseline and DAS28 at baseline as co-variates. Fig. 3A summarizes the results: 13 miRs were found to have significantly different changes in expression over the course of treatment between responders and non-responders, which were robust to multiple test correction, namely miR-761, miR-301b-5p, miR-548ar-3p, miR-212-3p, miR-188-5p, miR-410-3p, miR-661, miR-3130-3p, miR-1185-5p, miR-3180, miR-543, miR-105-5p and miR-338-5p. The contrasting dynamic expression changes are depicted for each of these in Fig. 3B (see also Supplementary Table S7, available at *Rheumatology* online). Of these, miR-301b-5p was the only miR to have a more positive change over time in non-responders than responders. Literature searches confirmed four of these miRs have been implicated in RA pathogenesis, all of them through interactions with synovial fibroblasts. First, miR-212-3p regulates the pro-inflammatory phenotype of these cells via inhibition of SOX5 (SRY-box transcription factor 5), whilst miR-338-5p has been suggested to have a cytostatic effect via inhibition of ADAMTS-9 (a disintegrin and metalloproteinase with thrombospondin motifs 9) [36, 37]. MiR-410-3p has also been shown to restrain proliferation of these cells whilst regulating their migration and pro-inflammatory cytokine release [38, 39]. Finally, a cytostatic effect of miR-537 on synovial fibroblasts has also been suggested [40]. Intriguingly, of the 13 miRs identified, five also exhibited a MTX response–age–sex interaction. In summary, known functions of miRs dynamically upregulated in circulating EVs of MTX-responders converge on their potential to restrain fibroblast-mediated pathology.

## Discussion

Our report describes, to our knowledge, the first comprehensive evaluation of miR cargo in circulating EVs of newly presenting RA patients naïve to immunomodulatory therapy; it furthermore appraises dynamic changes in EV miR expression during the first 4–6 months of MTX therapy, linking them to clinical response to this routinely used, first-line intervention. In presenting it we introduce an innovative platform for biomarker discovery applied in a highly relevant clinical setting whilst shedding light on disease pathogenesis and mechanisms of MTX efficacy in early RA.

We first identified a subset of 28 miRs whose expression patterns in circulating EVs may have value in discriminating untreated RA patients from individuals referred to a secondary care rheumatology department without evidence of a rheumatic disease as well as those presenting with an alternative IMID (PMR); this could conceivably contribute to a diagnostic tool in the future. There have been a number of reports of differential EV miR expression in relevant settings that provide some context to our observations. For example, Xu *et al.* highlighted robust downregulation of miR-6089 and miR-548a-3p in RA patients compared with healthy donors, with miR-6089 apparently regulating toll-like receptor 4 (TLR4)-mediated lipopolysaccharide production in myeloid cells [41]. In other work, ‘remarkable’ differences in miR profiles between RA and healthy donor-derived exosomes has been reported, including upregulation of miR-17 (which may inhibit regulatory T cell differentiation), miR-19b, and miR-121, and downregulation of miR-584a-3p (which may regulate the TLR4–nuclear factor-κB axis) [42, 43]. Chen *et al.* have published a list of 36 miRs differentially expressed in RA patients compared with healthy donors that were similarly deregulated in other inflammatory conditions—including psoriatic arthritis, psoriasis vulgaris and gouty arthritis [44]. Of these, we found only miR-370-3p to be deregulated in our dataset. Importantly, however, all of these comparisons were between patients with established RA (receiving treatment) and healthy individuals, meaning that differential miR expression attributable to systemic inflammation alone—or indeed background disease modifying therapy—cannot be excluded; careful measures to control for these effects (along with the potentially confounding impact of age and sex on miR expression [45]) are a strength of the current study and could very well explain why the aforementioned findings are not corroborated by our own [46]. Through deployment of the NanoString nCounter platform for low concentration EV miR profiling using recently validated methodology [15], as opposed to traditional array/real-time PCR technology employed by the referenced studies, we were also able to circumvent the need for amplification or cloning steps during RNA preparation as a potential error source. Indeed, the precipitation of EVs is increasingly seen as the most appropriate method for biomarker studies in this context [47]. Our observation of significantly reduced miR-337-3p cargo in circulating EVs of early RA compared with control groups, and the potential implications in terms of enhanced Janus kinase–signal transducer and activator of transcription signalling (which we and others have shown is a characteristic feature of this disease) is of interest, along with any putatively ‘pro-inflammatory’ consequence of the early RA profile in general [31, 48]; whilst clearly in need of independent/mechanistic validation, the concept proposed by other investigators that EV profiling may yield insight into pathogenesis as well as biomarkers *per se* is, in our view, thereby broadly reinforced [7].

Next, focusing solely on early RA patients, all of whom were commenced on MTX and clinically well-characterized during the first 6 months of treatment, we explored serum EV miR content as a predictive and/or potential mechanistic biomarker of early remission induction by this intervention. With regards to *prediction*, our cross-sectional analysis of baseline samples was underpowered; given the clinical and molecular heterogeneity of RA alongside a long list of proposed molecular biomarkers that have failed to translate to clinically valuable discriminators to date, we do not find this altogether surprising. Rather, we consider the results of PLS-DA (Fig. 2B) as proof of principle that circulating EV miRs could, weighted alongside promising readouts from other platforms, contribute a valuable component to a ‘liquid biopsy’ for application in newly diagnosed disease that enables more personalized therapeutic decision-making in the future [49]. Of relevance here, recently emergent data from the UK Rheumatoid Arthritis Medication Study (RAMS) initiative have consistently emphasized the added value of short-term MTX-induced *perturbations* in molecular parameters (determined through measurements before and shortly after drug initiation)—over single, pre-treatment ‘snapshot’ assays—as putative biomarkers of therapeutic efficacy over the longer term [50].

Interpreted in this light, we consider our observations in relation to differential miR regulation over time in response to MTX initiation between responders and non-responders (Fig. 3) to be of particular importance. On one level, they are consistent with the possibility that, by bringing the second serum EV miR measurement forwards (i.e. to 4 weeks from baseline as opposed to the median 18 weeks employed in our protocol), an added *predictive* utility to the bioassay comprising the 13 miRs highlighted might emerge, which could support early treatment augmentation amongst individuals unlikely to experience subsequent drug-induced remission by 6 months. Whilst this cannot be confirmed currently due to our study’s design, the possibility represents a readily testable priority for future work. On another level, our findings raise a tantalizing hypothesis in relation to *mechanism* of MTX efficacy: based on the increased abundance amongst MTX responders of EV miR content suggested to modulate pathogenic functions of synovial fibroblasts (miR 212-3p, miR-338-5p, miR-410-3p and miR-537), we speculate MTX could exert its effect in part through increased production and transport of these miRs to the synovium to exert anti-pathogenic effects [36, 38, 41], although this together with their cellular origin requires elucidation. Our observation that, of these eight candidates, five show interactions with age, sex or both is furthermore consistent with well-described associations of demographic factors with treatment responses seen in the clinic.

Strengths of our study include implementation of a robust, innovative and validated protocol for serum EV miR profiling that lends itself well to biomarker applications, amongst well-characterized clinical cohorts in a relevant context and with due mitigation against potential confounding factors. Findings from our relatively small and exploratory investigation must, however, still be viewed as preliminary, in need of independent validation and mechanistic study. Nonetheless, in addition to proposing measurable components of circulating EV cargo with potential as diagnostic and/or theragnostic tools in early RA, our work highlights a biologically plausible, fibroblast-mediated mechanism by which MTX may exert its therapeutic effect that warrants further investigation in functional evaluations.

## Supplementary Material

kead569_Supplementary_Data

## Data Availability

The data underlying this article are available in the article and in its online supplementary material.
