# Supplementary material for: Micro-RNA content of circulating extracellular vesicles in early rheumatoid arthritis as biomarkers and mediators of methotrexate efficacy
Source: Rheumatology (Oxford). 2023 Nov 1;63(8):2259–67. doi: 10.1093/rheumatology/kead569 (PMC11292053; doi:10.1093/rheumatology/kead569)
Supplement: kead569_Supplementary_Data [file kead569_supplementary_data.zip › kead569_Supplementary_Data/rhe-23-1198-File005.docx]

**Micro-RNA content of circulating extracellular vesicles in early rheumatoid arthritis as predictive biomarkers and mediators of methotrexate efficacy.**

Maunder et al.

**Supplementary File.**

**Supplementary Data S1. Supplementary Methods.**

In relation to serum EV isolation, miR extraction and quality control, additional information is provided here; the reader is also referred to Reference 13 (Crossland RE *et al*) in the main manuscript.

Serum aliquots were further centrifuged at 4,500×*g* for 15 min, and EVs were isolated from a minimum of 1.5 ml of serum using Total Exosome Isolation Reagent (ThermoFisher Scientific) following supplier guidelines. Total RNA was isolated using Total Exosome RNA and Protein Isolation Kit (ThermoFisher Scientific) following supplier’s protocol. RNA was concentrated incorporating Amicon Ultra-0.5 centrifugal filter unit with Ultracel-3 membrane (Merck Millipore), according to NanoString recommendations. RNA quantity was determined using the 2100 Bioanalyzer and the RNA 6000 Pico Kit (Agilent Technologies). All RNA was stored at −80°C.

For EV characterisation, morphology and size distribution were assessed [16]. For EV morphology, transmission electron microscopy (TEM) was performed using 300-mesh grids, filmed with Pioloform® resin (SPI Supplies), carbon coated, and plasma etched before use. EVs were directly applied to the grid, stained with 10 *μ*l of 2% aqueous uranyl, and air-dried. Examination was conducted on a Hitachi HT7800 transmission electron microscope with an Emsis Xarosa camera and Radius software.

For EV size distribution analysis, nanoparticle tracking analysis (NTA) was performed employing a NanoSight LM10 microscope supplied with NTA software version 3.2 (NanoSight Ltd., UK). Background extraction with blur settings and maximum jump distance was applied automatically, and 5x60 second recordings were taken for each sample

**Supplementary Figure S1.**


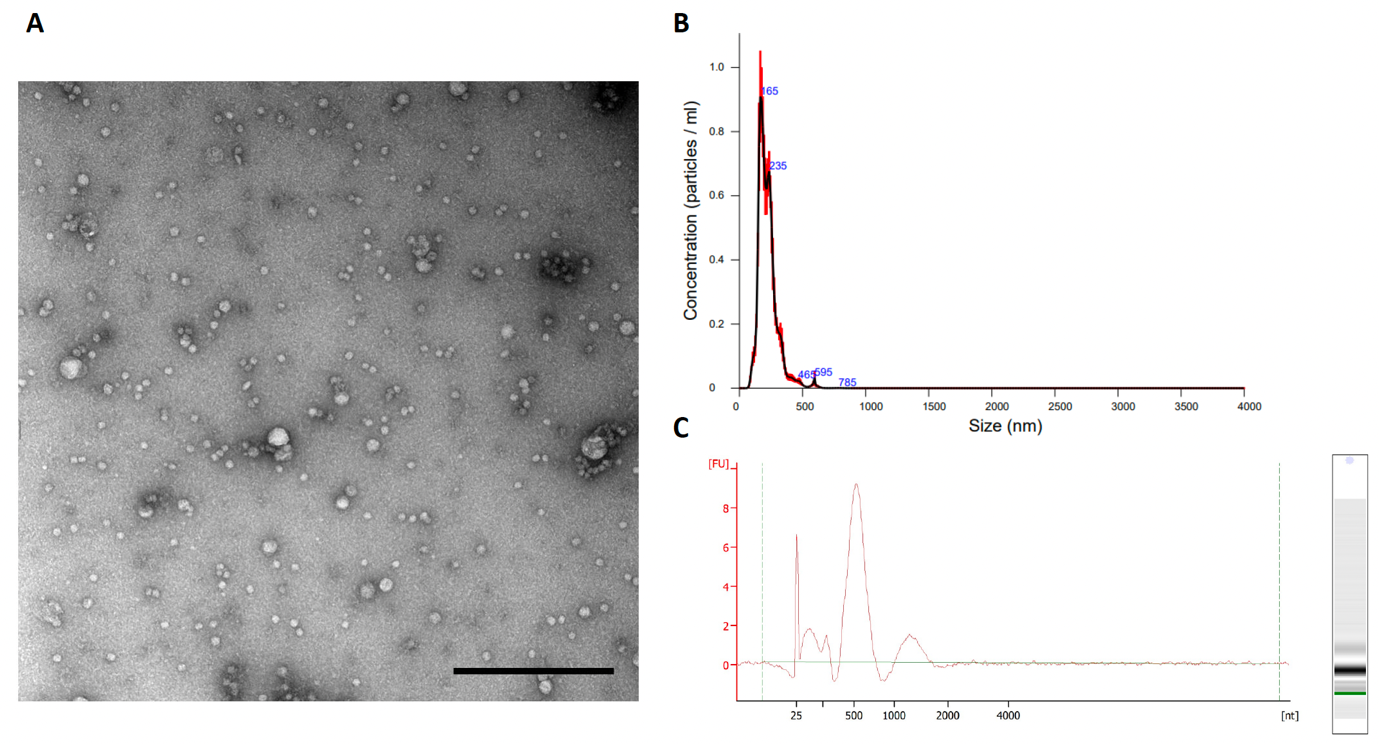


***Supplementary Figure 1. A.*** *A representative electron micrograph of visualised vesicles isolated from a single serum sample. Scale bar=500nm.* ***B.*** *NTA by NanoSight LM10 microscope (5 captures) indicating a mode EV size of 175nm.* ***C.*** *An exemplar electropherogram showing RNA concentration in isolates from EVs.*
